# Salt Stress—Regulation of Root Water Uptake in a Whole-Plant and Diurnal Context

**DOI:** 10.3390/ijms24098070

**Published:** 2023-04-29

**Authors:** Yingying Lu, Wieland Fricke

**Affiliations:** School of Biology and Environmental Science, University College Dublin (UCD), Belfield, D04 N2E5 Dublin, Ireland; yingying.lu@ucdconnect.ie

**Keywords:** aquaporin, Casparian band, endodermis, night-time transpiration, plasmodesmata, suberin lamellae, salinity, xylem tension

## Abstract

This review focuses on the regulation of root water uptake in plants which are exposed to salt stress. Root water uptake is not considered in isolation but is viewed in the context of other potential tolerance mechanisms of plants—tolerance mechanisms which relate to water relations and gas exchange. Plants spend between one third and half of their lives in the dark, and salt stress does not stop with sunset, nor does it start with sunrise. Surprisingly, how plants deal with salt stress during the dark has received hardly any attention, yet any growth response to salt stress over days, weeks, months and years is the integrative result of how plants perform during numerous, consecutive day/night cycles. As we will show, dealing with salt stress during the night is a prerequisite to coping with salt stress during the day. We hope to highlight with this review not so much what we know, but what we do not know; and this relates often to some rather basic questions.

## 1. Plants and Salinity

### 1.1. Global Significance

Over 6% of the world’s land area (around 800 million hectares) suffers from excessive salt concentration. This severely affects the germination, growth and productivity of crops (FAO, 2005 Global Network on Integrated Soil Management for Sustainable Use of Salt-affected Soils. Rome, Italy: FAO Land and Plant Nutrition Management Service. http://www.fao.org/ag/agl/agll/spush, accessed on 15 March 2023). Most salt-affected soil originates from natural causes, such as the accumulation of salt in arid and semiarid areas over time, or the deposition of oceanic salts carried by wind and rain. An increasing and significant portion of salt-affected land has resulted from agricultural practices involving land clearing and irrigation, which leads to a rise in groundwater tables and causes secondary salt to accumulate in root-zone groundwater [1]. Climate change with associated global warming has not helped either. Unfortunately, most crop plants are glycophytes. For example, a field concentration of 100 mM NaCl will kill salt-sensitive rice cultivars before they mature and set seeds, and decrease yield in moderately salt-tolerant wheat [2]. Even barley, the most salt-tolerant species among our cereal crops, dies after extended periods of exposure to 250 mM NaCl [3]. One could argue that the by-chance discovery of a barley or wheat genotype which yields 100% at say 300 mM NaCl may just delay the problem, by relieving the pressure of the need to change agricultural practices, but there is no doubt that it would help to safeguard future food supply in many areas in the short term. Identifying stress tolerance mechanisms which enable plants to yield under salinity will help to achieve this in a more targeted approach. The first step is to ask which potential stresses salinity causes to plants, and which basic options plants have, in theory and practice, to tackle those stresses. We are focusing here on salt stress in a humid setting, where the dominant salt is sodium chloride, rather than as in dry, e.g., steppe regions, and carbonates of sodium or calcium. In the latter setting, salinity is mostly associated with an alkaline soil pH, which may cause as much or more stress to plants than the actual salt concentration [4].

### 1.2. The Major Stresses Associated with Salinity

**Figure 1:**
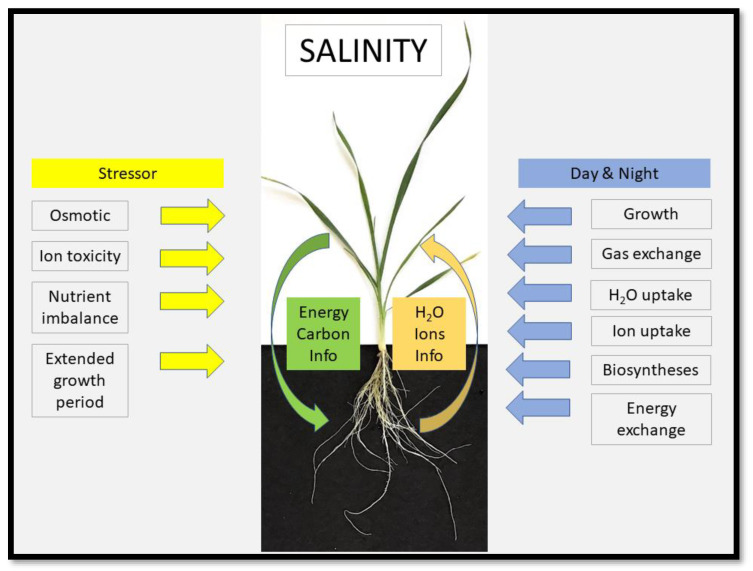
Salinity causes some major stresses to plants. The two most obvious stresses are osmotic stress, due to the accumulation of salt in the root medium, and an ion-specific toxicity stress. Ion-specific stress is due to Na^+^ and Cl^−^ in humid environments, where salinity is caused by NaCl. Carbonates of Na^+^, Ca^2+^ and Mg^2+^ are the major salts in dry environments, such as steppe regions, and cause the pH of soil to becomes alkaline. A further stress caused by salinity is mineral nutrient imbalance, as salt competes with the uptake of minerals such as K^+^ and NO_3_^−^. Plants can compensate to some extent for slower growth rates during stress through longer growth periods. Such a compensatory mechanism is limited in annual plants, where growth is restricted to a certain period of year. This causes another potential stress due to salinity. Salt impacts during day and night on a range of physiological processes: these are growth, gas exchange, water and ion uptake, biosynthesis and the acquisition and expenditure of energy. The image shows a three-week old wheat plant, highlighting the interaction and resource exchange between shoot and root.

The two main, direct stresses caused by salinity to plants are (i) osmotic stress/water deficit stress and (ii) ionic stress [1] (Figure 1). A third, associated stress is (iii) mineral nutrient imbalance, as Na^+^ and Cl^−^ interfere or compete with the uptake of mineral nutrients such as K^+^, Ca^2+^ and NO_3_^−^ (Figure 1). The high salt concentration (lower osmotic potential) in the soil lowers the water potential (ψ) and makes it harder for plants to take up water. Continuous uptake of NaCl by the root system and delivery to the shoot with the transpiration stream builds up high concentrations of NaCl within leaf cells. This will ultimately inhibit leaf biochemical processes, particularly photosynthesis, and lead to a nutrient imbalance [5,6,7,8]. Conserving water and reducing the loading rate of the leaf with salt can both be achieved through reducing leaf gas exchange by stomatal closure, yet this also reduces carbon assimilation and energy fixation. Finding the best possible compromise between these opposing needs is the challenge to plants which grow in a saline root environment. A fourth stress which can be caused by salinity is not so much related to the nature of the stressor, but more related to the resulting retardation of growth and development. This applies in particular to settings, and annual crop plants, where the climatic ‘window’ during which crops can be grown is time-limited to a certain period during the year. Under these conditions, stressed plants may not be able to make up for reduced growth rates by merely growing for longer periods (Figure 1).

#### 1.2.1. Osmotic Stress

Plants which develop in the field on a saline substrate experience osmotic stress throughout their lifetime as a type of underlying stress component of salinity. They will not experience any osmotic shock, as would occur in a laboratory environment when salt is added rather suddenly to the soil or nutrient solution in which plants grow. The extent of osmotic stress in a field setting varies throughout a 24-h day/night cycle, with stress being most severe during the day and least severe during the night. Cell ψ, turgor and osmotic adjustment may vary in parallel, but any changes in these sizes occur rather gradually when compared to changes when plants are exposed to salinity rather suddenly in a laboratory setting. In the latter, plants show a temporary and rapid response to the osmotic stress component of salt stress [9]. The response starts within seconds to minutes and can be completed in as little as a few hours. Leaf cell turgor and elongation rate rapidly decrease within minutes, irrespective of the nature of the salt, as the same response would appear when KCl, mannitol or polyethylene glycol (PEG) were added at equivalent osmotic pressure. Root growth slows down as well [10]. To which extent these reductions in growth remain or can be recovered depends on the ability of the cells to lower ψ equivalent to the reduction in root medium/soil ψ while not compromising their biochemical activity. Mature cells have only one option to achieve this, and this is through osmotic adjustment, the net accumulation of solutes in cells so that turgor recovers to or remains at the unstressed control level [11]. Growing, expanding cells, in contrast, have the additional option to alter the properties of their walls, to ‘soften’ walls. This enables expansion at a reduced turgor as walls mechanically yield and assures that the ψ of growing leaf cells can be lowered without the immediate need to net accumulate solutes. This approach may seem rather elegant, yet it just ‘borrows time’: the net accumulation of solutes is in the longer term the only means through which the newly-formed, mature cells can maintain turgor and the ψ difference to root medium as one would observe under non-saline, control conditions. Given the above, it is not surprising that osmotic adjustment is currently experiencing some revival in the literature as a neglected tolerance mechanism to drought and osmotic stresses [12,13].

Osmotic adjustment and turgor regulation are processes which relate to the adjustment of ψ at the cellular, protoplasmic level. The other major compartment within plants is the cell apoplast. The apoplast comprises the wall space, middle lamellae and intercellular air space of tissues. It also includes the lumen space of xylem conduits (vessels, tracheids). The later space is ‘special’ in that it contains water mostly in a metastable state, as the hydrostatic pressure in the xylem is significantly below atmospheric, particularly during the day [14]. Water in wall spaces is instead in a more stable state due to the capillary forces which act here in the comparably much finer micro- or nano-channels within the wall matrix. Lowering ψ in the root medium through the presence of salt lowers ψ in the apoplast, and one large potential stressor is that ψ and the tension in the xylem become so negative that cavitation occurs. The other large potential stressor applies to the wall space of living cells. The volume of water in this wall space is small. That means that it does not require much salt to accumulate here for the osmotic pressure to increase, and ψ to decrease. This can lead to a partial dehydration and turgor loss of the cell protoplasm (Oertli hypothesis) [15].

#### 1.2.2. Ion Toxicity

**Figure 2:**
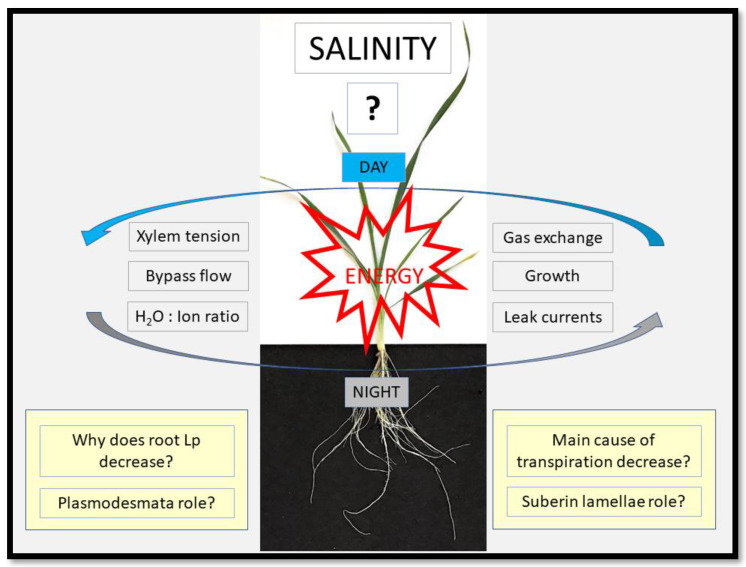
We know hardly anything about the relevance of night-time processes and their interaction with day-time processes for the tolerance of plants to salt. Processes which are of interest here and which have to continue or are dealt with/regulated throughout day and night are xylem tension, bypass flow, water-to-ion uptake ratio, gas exchange, growth and leak currents of ions. This all requires energy. Although it is well documented that root hydraulic conductivity (Lp) and transpirational water loss decrease in response to salt stress in e.g., crop plants, we need to ask why these decreases occur. The answer to these questions seems obvious, but is it really (see main text)? We also need to ask what the role of plasmodesmata is in roots to facilitate a high water-to-ion uptake ratio, and which role suberin lamellae have in this process, and also in the avoidance of embolism formation in a xylem under increasingly high tension. The image shows a three-week old wheat plant.

Salt-specific stress, or ion toxicity, is due to the excessive accumulation of Na^+^ and Cl^−^ in plant tissues, particularly transpiring leaf tissue as the ‘endpoint’ of the transpiration stream. As Na^+^ and Cl^−^ continue to build up in cells, they ultimately exceed the capacity of cell to compartmentalise these ions in the large central vacuole—the space within cells which is the least metabolically active and the least discriminate in terms of which ions are required at which concentrations [16,17]. Ion concentrations then increase to levels in the cytoplasm (cytosol, plus cell organelles except the nucleus) where the biochemical activity and structural integrity of proteins is compromised. Cell metabolic rate, membrane function and cell viability decrease. The larger the ratio between the vacuolar to extra-vacuolar space within a cell, the more salt arriving in a cell can be accumulated in the vacuole. Also, fewer alternative solutes need to be used to osmotically counterbalance the cytoplasm. In other words, the vacuolar to extravacuolar volume ratio is one key feature of cells which affects the susceptibility to ion toxicity. This ratio differs substantially between different tissues, particularly in leaves (Figure 2). Mesophyll cell volume is roughly half occupied by the vacuole, whereas epidermal cells often have 95% and more of their volume vacuolar [11,18]. Cells which are newly produced within meristems do not have a central vacuole at all, but instead a conglomerate of many smaller vacuoles (‘vacuon’). The cellular volume proportion of the vacuole increases as a cell expands to their final, mature volume. It follows that meristematic cells and cells early on during expansion are potentially the most susceptible to ion toxicity, whereas epidermal cells in the mature blade are the least susceptible. Meristematic and growing tissues either do not transpire or transpire very little. The ion load which arrives with the transpiration stream within these tissues is small compared to that which arrives in the mature leaf blade. This reduces the potential ionic stress experienced by meristematic and growing leaf tissues. It is surprising how few studies which look at tolerance mechanisms to salinity distinguish between growing, mature and meristematic and also between mesophyll and epidermal leaf tissues [13,19,20,21,22,23,24]. The weakest link in this chain of tissues will determine the overall salt tolerance of a species. Other factors contribute to potential ion toxicity. For example, exposure to salt of a salt-sensitive crop for serval days will cause visible salt-specific effects, such as yellowing of the leaf or dead old leaf. Survival of the plant then depends on the relative death rate of the old leaf and the production rate of new leaf tissue [3]. If an adequate number of green leaves is left to supply the necessary photosynthate, the plant survives to the reproductive stage. For most species, Na^+^ appears to reach a toxic level faster than Cl^−^, and most studies have focused on how plants can avoid Na^+^ toxicity [1]. For some species, notably soybean, citrus and grapevine, Cl^−^ causes more ion toxicity [1]. 

#### 1.2.3. Nutrient Imbalance

External Na^+^ and Cl^−^ often competitively inhibit the uptake of other minerals. This can cause symptoms of mineral deficiency. The mineral elements in question include boron (B), zinc (Zn), calcium (Ca), copper (Cu), magnesium (Mg), iron (Fe), nitrogen (N), phosphorus (P) and potassium (K). For example, a decreased ratio of K^+^ to Na^+^ was observed in salt-stressed chickpea [25], faba bean [26] and mung bean [27], resulting in K^+^ deficiency and significant yield reduction [28]. Potassium is involved in numerous metabolic processes, and it is generally assumed that the cytosolic K^+^ concentration should ideally be between 60–100 mM [16,23]. This is the highest concentration range of all mineral nutrients, and the ability to maintain a high K^+^/Na^+^ ratio in the cytosol, and cytoplasm, is thought to be a key trait associated with salt tolerance in many species (wheat, barley, rice, Arabidopsis) [29,30,31,32,33,34,35,36]. There exist numerous membrane transport systems for K^+^, and many of these show a much higher selectivity for K^+^ compared with Na^+^ [37,38,39,40]. However, even a selectivity ratio of say 100 K^+^ to 1 Na^+^ will lead, in a soil environment with 1 mM K^+^ and 200 mM Na^+^, to the uptake of twice as much Na^+^ compared with K^+^. This explains in part why it may be so difficult for plants to minimise their uptake of Na^+^ in a saline root environment. The interference of Cl^−^ with the uptake of NO_3_^−^ has received comparatively little attention, even though it can lead to reduced uptake and assimilation of N [41]. Low levels of Cl^−^ may be beneficial for the growth of plants, not just for halophytes, but at a lower concentration range also for glcyophytes. Nitrate fulfils an important osmotic function in the vacuole of cells. It is the main inorganic anion which counterbalances cationic ions such as K^+^, Na^+^, Mg^2+^ and Ca^2+^. The osmotic function of NO_3_^−^ can be taken over by Cl^−^ [42]. This frees up NO_3_^−^ for additional assimilation of N, an aspect which has received surprisingly little attention despite its agronomic potential, also in a changing climate.

### 1.3. Salt Tolerance

If we were to design an ideal plant which can cope with the osmotic, ion toxicity and nutrient imbalance stress of salinity, we would opt for a plant which can:

- Osmotically adjust cell water potential (ψ) so that the change in cell ψ (Δψ) equates to Δψ in the root medium; maintain cell turgor at the level observed for unstressed plants;
- Tolerate xylem tensions, which increase by Δψ, without embolism;
- Osmotically adjust through the use of Na^+^ and Cl^−^ in the large central vacuole, and through use of alternative solutes in the cytoplasm (and nucleus), which are compatible with cell functions (‘compatible’ solutes);
- Take up only as much Na^+^ and Cl^−^ as required for osmotic adjustment in the vacuole;
- Maintain leaf gas exchange and root water uptake at the level of non-stressed plants;
- Maintain a growth rate so that growth and any seed production can be completed within the usual time frame;
- Maintain the uptake of mineral nutrients, particularly K^+^, Ca^2+^, Mg^2+^ and NO_3_^−^ at the level of non-stressed plants;
- Do the above during day and night.

One could argue that we are asking our ideal plant not to do any magic but to apply processes which operate daily. For example, the endodermis has ion-filtering properties due to suberin lamellae and, particularly, Casparian bands [43,44,45,46]. These filtering properties could be optimised to keep Na^+^ and Cl^−^ levels in the xylem and the ion load of leaves at the required low levels while supplying the shoot with water at the usual rate. This would allow leaf gas exchange including carbon assimilation to proceed as usual. A stress with 200 mM NaCl will lower soil ψ by almost 1 MPa and in our case, increase xylem tension by 1 MPa. This may still not exceed tensions which cause embolism, nor does it have to lower leaf ψ past the permanent wilting point in many crops (about −1.5 MPa; [4]). Osmotic adjustment in the cytoplasm can be achieved through a range of compatible, organic, solutes. These include proline, glycine betaine, b-alanine betaine, choline-O-sulfate, hydroxyproline, dimethylsulfonium propionate (DMSP), and putrescine [47,48,49] (Hanson et al., 1994; Summers et al., 1998; Bouchereau et al., 1999; Hu et al., 2015), and polyols such as glucosylglycerol, glycerol, mannitol, myo-inositol, ononitol, pinitol and sorbitol [50,51,52,53,54,55,56,57,58] (Gorham et al., 1980, 1981; Vernon and Bohnert, 1992; Ishitani et al., 1996; Murakeozy et al., 2003; Arndt et al., 2004; Koyro, 2006; Stoop et al., 1996; Noiraud et al., 2001). Even if Na^+^ was leaking into the root stele, plants have mechanisms to reduce the load of Na^+^ which arrives in the leaf with the transpiration stream thanks to ion transport mechanisms in parenchyma cells of the root xylem, stem and leaf sheath [59] (Farquharson, 2009). For example, in wheat, Nax2 (TmHKT1;5-A) [60,61,62] and Kna1 (TaHKT1;5D) [63] which are expressed in root stelar cells limit the amount of Na^+^ that is transported in the xylem to the leaf tissues, while retrieval of Na^+^ into the leaf sheath is a trait conferred by Nax1 (TmHKT1;4-A2) and limits Na^+^ accumulation in leaves [60]. In *Arabidopsis*, overexpression of the Na^+^ transporter AtHKT1;1, which is expressed in root stele and leaf vasculature, leads to increased shoot Na^+^ exclusion and increased salinity tolerance [64,65,66]. The plasma membrane Na^+^/H^+^ antiporter AtSOS1 is expressed in epidermal cells at the root tip and in parenchyma cells at the xylem/symplast boundary of roots, stems, and leaves, and has been proposed to re-extract Na^+^ from the xylem under salt stress condition [67]. If turgor is maintained at the control level, expansion of root and leaf cells and the associated increase in root and shoot surface area should proceed as well. So, why is it then, that our ideal plant remains elusive in the real world? The answer can be grouped into five aspects. 

Firstly, the plant does not live in isolation in its natural environment, and there exist processes which are outside the control of the plant. This applies in particular to the saline root environment. Continuous exclusion of salt at the root surface—and at the root endodermis—combined with the convective movement of salt with water toward the root surface, leads to the build-up of salt concentrations which become increasingly higher compared with the salt concentration in the bulk soil environment. Thus, the better plants exclude salt at the root surface, and the more convective movement of water and salt occurs, the lower ψ becomes at the root surface. To still take up water, plants will have to lower leaf ψ and increase xylem tensions more than predicted based on bulk soil ψ. It is in many ways a no-win situation.

Secondly, there are physicochemical, or physical, limits to how metastable water can be in the xylem, and how negative xylem tensions can become during peak day times such as midday, without risking embolism [14].

Thirdly, cells in growing root and leaf tissues expand to a multiple of their original volume. Although these cells have a vacuole-to-extravacuole ratio which is small compared with the ratio in mature tissue, they do continuously dilute incoming salt. As long as a cell expands, salt dilution minimises the detrimental effect of salt on cell metabolism, similar to salt succulence in mangroves [56,68]. In contrast, cells in mature root and more so in mature leaf tissues have reached their final volume, and salt dilution is not an option. Even worse, mature leaf blades are the main source organs, and rates of gas exchange with associated transpirational water loss are highest here. Keeping the salt load in these tissues low over time, while guaranteeing the usual lifetime of blades would require additional mechanisms, such as salt glands [69,70] to get rid of the excess salt. An alternative would be to have xylem ion concentrations specifically and manyfold lower in vessels which supply mature as opposed to growing leaf tissues. It is difficult to imagine how this could work given the continuous nature of xylem architecture in a plant [14,71].

Fourthly, and this is maybe the most important aspect, all the processes which are carried out by our ideal plant cost carbon and energy. This applies to e.g., membrane transport processes, dealing with leak currents across membranes, synthesising compatible solutes or rendering apoplastic barriers less leaky. The amount of carbon and energy invested in those processes comes on top of that needed to grow and yield at unchanged rates. Additional carbon and energy can only be obtained through an increase in the net rate of photosynthesis. While we do not know the precise amount of additional carbon and energy required, we can assume that this amount is substantial [72,73]—too substantial for any additional transpirational water loss to be met by compensatory increases in water use efficiency (WUE). Thus, the most likely scenario would be that root water uptake has to increase. Increasing root water uptake through an increase in the driving force, xylem tension, is a risky strategy due to potential embolism at peak times of water consumption. Increasing root water uptake through an increase in the root water transport properties (hydraulic conductivity, Lp; rate of water uptake per unit root surface and driving force; unit m^3^ m^−2^ s^−1^ MPa^−1^, or m s^−1^ MPa^−1^) is the only alternative option. However, root Lp seems to operate already at its maximum capacity under non-stress conditions and, if anything, decreases in response to abiotic stress [74]. We are left with another catch-22: assimilate more carbon and energy to support the adaptive mechanisms in our ideal plant, but do not increase the rate of transpirational water loss significantly.

Finally, we need to consider the night period. Biochemical activity during the night period depends on carbon and energy resources acquired and stored during the day. Plants also grow in size and transpire water during the night [75,76,77]. How much water is transpired and how much shoot area expands—there is little known about roots [78]—depends on the species, genotype and environmental conditions [75]. Leaf area can expand at rates similar to or even exceeding rates during the day-time, while night-time water loss occurs often at 5–15% of day-time rates [77]. Leaf and root area expansion is supported through carbon which is fixed and supplied by source leaves during the day. The same applies during the night, except that carbon is made available through the degradation of storage carbohydrates (starch, fructans) accumulated during the day [79,80,81,82,83]). Studies, mainly on *Arabidopsis*, have shown that plants adjust night-time rates of growth to the availability of storage carbohydrates. This causes a tight functional link between day- and night-time growth and the provision of new photosynthetic leaf area [84,85]. If our ideal plant grew at unchanged rates under salinity, it would have to achieve this during day and night. This in turn would require an unchanging amount of storage carbohydrates which can be consumed during the night. However, transport processes, synthesis of compatible solutes for newly generated cytoplasmic space and dealing with leak currents of ions out of the vacuole, or Na^+^ and Cl^−^ into cells, uses up some of this energy. Again, day-time photosynthesis and provision of storage carbohydrates would need to significantly increase. Having said this, plants are less likely to be hydraulically limited in growth during the night compared with the day [80,86,87]. Could it be that night-time transpiration and growth provide some adaptive mechanisms to plants under salinity [77]? We address this question in the next section, before focusing on the regulation of root water uptake in a diurnal context as well.

## 2. Night-Time Transpiration and Growth

An extensive body of research has assessed the magnitude and regulation of day-time transpiration while night-time transpiration has always been assumed to be negligible due to “stomatal optimisation”—plants maximise carbon fixation while minimising water loss. Based on this paradigm, there should be no transpiration occurring when net CO_2_ assimilation is impossible due to the lack of sunlight. Stomata should be closed then. However, incomplete stomatal closure during the night has been reported across a range of species among C_3_ and C_4_ plants and across a range of ecosystems [88,89], including tropical rainforests [90,91,92], temperate woodlands [93], Mediterranean forests [94], semi-arid woodlands [95,96], deserts [97] and managed systems including plantations [92,98], common gardens [99] and whole-tree chambers [100]. In addition, most species have the ability to close stomata more than is commonly observed at night, as demonstrated by reduced night-time leaf conductance in response to water stress or application of abscisic acid (ABA) [86,101].

### 2.1. Factors Which Affect Stomatal Conductance and Night-Time Transpiration

Day- and night-time water loss through stomata, and stomatal conductance, are in principle affected by the same factors, yet this does not imply that water loss during the night can be predicted based on information on water loss during the day. Factors which impact on stomatal conductance during the night are, in particular, vapour pressure deficit (VPD) [89,102,103], temperature [104,105,106], CO_2_ concentration [106,107,108,109], ABA [110,111,112,113,114], water availability [105,110,111,114,115,116], soil nutrient concentration [115,117], endogenous circadian clock [118,119,120], photosynthesis and light [121,122,123,124]. Atmospheric water demand is driven by a difference in vapour pressure and associated differences in water vapour concentration between the inside and outside of the leaf. While VPD is the major driver of day-time stomatal conductance, its effect during the night on transpirational water loss is less clear. For many tree species in a natural environment, increased VPD correlated with increased night-time transpiration at the scale of sap flux [89,125]. In contrast, some species showed a negative relationship between VPD and night-time transpiration [103], and some data indicate no clear response [121]. Night-time stomatal conductance is just as sensitive to water stress as day-time conductance. For example, *Hibiscus cannabinus* [105], *Pseudostuga menziesii* [114,126], and *Helianthus anomalus* [115] showed a lower rate of night-time transpirational water loss at decreased water availability. In wheat grown in a greenhouse, water stress treatment decreased night-time transpiration, and the same applied in *Helianthus* species [110,111]. Night-time leaf conductance decreases in response to salinity [77,127] and ABA [114]. In Arabidopsis, N limitation caused decreased night-time leaf conductance [88], whereas increased night-time leaf conductance was observed under N limited conditions in *D. spicata* and *Populus balsamifera subsp. Trichocarpa* [88], and no response to nutrient limitation was observed in *Helianthus* species [111]. Day-time leaf conductance generally decreases in response to elevated atmospheric CO_2_ concentrations. Responses observed for night-time leaf conductance are more varied. Some species showed an increased night-time leaf conductance under elevated CO_2_ concentration, for example, *Triticum aestivum* [107], *Eucalyptus camaldulensis* [108], *Ipomoea batatas* [107] and *Eucalyptus sideroxylon* [106]. Other species, however, showed a negative effect of elevated CO_2_ on night-time leaf conductance, e.g., *Solanum tuberosum* [107], *Eucalyptus tereticornis* [128], *Ricinus communis* [129] and *Arabidopsis thaliana* [109]. For many species, (endogenous) stomatal conductance gradually increases during predawn hours, regardless of whether plants are grown in a field environment or under greenhouse conditions. For example, stomatal conductance at night rose slowly at predawn [120], a response which could not be observed in *Arabidopsis* mutants with disrupted circadian rhythm [118,119].

### 2.2. Why Do Plants Lose Water during the Night When No Carbon Can Be Gained?

Transpirational water loss during the night, which may be associated with partially open stomata, can have different reasons, or functions. These functions are related to the uptake and root-to-shoot delivery of mineral nutrients [130,131,132], hydraulic redistribution [55,133], the release of respiratory CO_2_ at sufficiently-high rates [75,131,134], potential benefits to day-time carbon gain [88,89,108,135], removal of xylem embolism and capacitance [96,136]), and a background permeability of the cuticle to water (for discussion, see [134]).

### 2.3. Does Night-Time Transpirational Water Loss Benefit Plants under Salinity?

Salinity causes a reduction in the rates of CO_2_ assimilation, transpiration and leaf expansion during the day in many crops [1] (Munns and Tester, 2008). There exist few data from investigations into whether similar reductions occur during the night under salinity, and how water loss through stomata and cuticle are affected relative to each other. A less negative xylem ψ during the night compared with the day should make it easier for growing leaf cells to take up water. As a result, a higher portion of the water delivered to the shoot may be stored within plants through growth rather than being lost through transpiration [77]. Night-time transpiration can also cause significant tensions in the xylem which obliviate the need to accumulate large concentrations of solutes in the xylem to draw in water [77]. These tensions will still be smaller compared with tensions during the day period, and any salt crossing the endodermis into the stele along a bypass path [137] will be smaller in quantity as well. Thus, night-time transpiration through stomata could have the combined advantage that it enables a higher portion of the water taken up to be kept in the plant, while accumulating fewer salt ions per unit of water taken up. We concluded, in a recent study on salt-stressed wheat plants which were grown on hydroponics, that night-time transpiration occurs through stomata and most likely serves the purpose to enable respiratory CO_2_ associated with night-time leaf growth to escape sufficiently rapidly from leaves to avoid acidosis in the cells [77]. The study further led us to conclude that night-time transpiration was associated with significant tensions in the xylem and an increased portion of the water taken up to be stored through leaf growth. The latter increase became significant at the higher salt concentrations tested (150–200 mM). Growing wheat plants under high relative humidity (RH) during the night and reduced night-time water loss did not affect the growth response of plants to salinity. The overall conclusion of this work was that night-time transpiration neither provided an advantage nor a disadvantage in coping with salt stress. It remains to be shown to which extent this conclusion applies to other crop species and to plants grown in the field. One rather unexpected observation of this study was that the overall amount of water lost over a 24 h day/night (16 h/8 h) period was not affected by growing plants under conditions of reduced night-time transpirational water loss (high RH), as day-time water loss rates increased. This could indicate some coupling between the water loss occurring during day and night, in a sense that plants can ‘measure’ the amount of water lost during each period. They could achieve this at a set water-use efficiency by ‘measuring’ assimilated (day) and lost (night) carbon.

## 3. Root Water Uptake

### 3.1. Roots Are Modular, Diverse and Highly Dynamic

The biophysical properties of roots, including their water and ion transport properties, depend to some extent on their morphology and anatomy. Species can differ considerably not only in root morphology, but also in the anatomy and (developmental) modular elements along the length of a single root, such as root cap, apical meristem, elongation zone, differentiation zone and mature zone [138]. Examples are the formation and development of aerenchyma, exodermis, endodermis with Casparian bands, suberin lamellae and thickened inner tangential walls, root cortical senescence (RCS), epidermal cell death, lateral root development, and the sclerification of older parts of the root system [139,140,141,142,143,144,145,146].

Plant roots provide anchorage in the substrate, they take up, store and translocate minerals and water, communicate with above-ground organs, and are at the interface between the plant and abiotic and biotic root environment, including the microbiome [147,148]. Variations of radial and axial water transport properties (hydraulic conductivity, Lp) result from differences in root maturation, membrane permeability (in terms of aquaporins), xylem vessel size, and vessels [149]. Therefore, root water uptake does not only vary between individual roots but also differs between different root orders and root types. Roots show high developmental plasticity and often adapt to their environment, which can affect their hydraulic properties as well, as will be discussed in the next section.

### 3.2. Root Water Uptake—Transport Paths and Driving Forces

**Figure 3:**
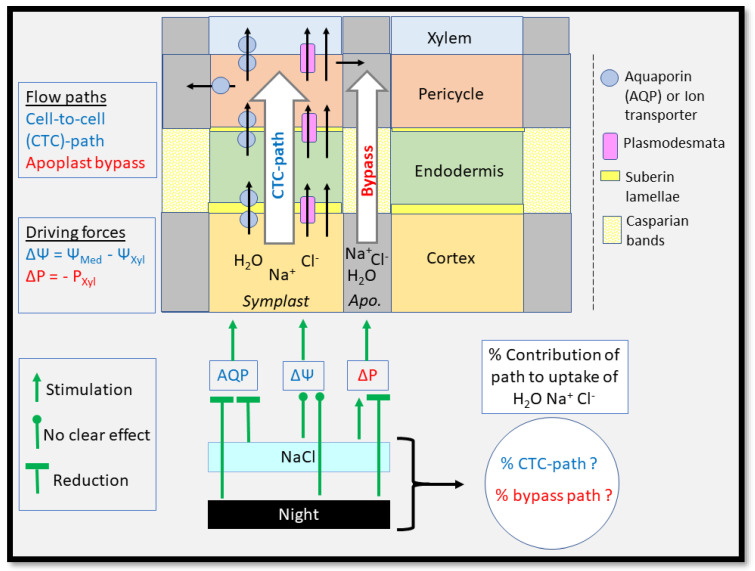
Different flow paths contribute to the movement of water and ions across the root cylinder. The flow paths shown here cover the root cortex, endodermis, pericycle and xylem, which is contained within the root stele; the epidermis is not shown. Water and ions, or any other solutes, move across two flow paths which are principally different. One path is the cell-to-cell (CTC) path. Substances can move here through either, or a combination, of: (i) transport proteins, or channels, for example through aquaporins (AQPs) in the case of water; (ii) plasmodesmata; or (iii) simple diffusion across the lipid bilayer of the plasma membrane. The alternative flow path is the apoplast path. Water and ions which move along this path bypass the cell protoplasm (‘bypass flow’), or in the case of many cells being connected through plasmodesmata, continuous symplasm. Flow along the apoplast is slowed down by the presence of so-called ‘apoplastic barriers’. These are typically suberin lamellae and, particularly, Casparian bands. Note, that the scheme shows only the part of the Casparian bands which is deposited in the radial walls of the endodermis; it does not show that the Casparian bands encircle the entire cell, that is why they are called ‘bands’. Suberin lamellae are deposited between the plasma membrane and wall of endodermal cells, particularly the peripheral walls. That means that suberin lamellae do not just slow down apoplastic flow but also impact potentially on the flow of substances along the cell-to-cell path between the innermost cortex layer and the endodermis. Suberin lamellae will not affect transport through plasmodesmata. Water and ions which are contained within the protoplasm of pericycle cells and move into the xylem can do this either along the usual CTC route, or simply by exiting through the plasma membrane (lipid bilayer, transporters/channels) of the pericycle cell into the apoplast of that cell, which is continuous with the apoplast of the xylem. The two flow paths also differ in the biophysical forces which drive the flow. The driving force is given here just for water; ions move along an electrochemical gradient or can be moved under expenditure of energy. A difference in water potential (ΔΨ) between the water potential in the root medium (Ψ_Med_) and xylem (Ψ_Xyl_) drives the flow of water along the CTC path. The apoplast is assumed to have no semipermeability, and osmotic forces cannot act here. Instead, mass flow along the apoplast is driven by a difference in hydrostatic pressure (ΔP) between the root medium and xylem (P_Xyl_). The pressure in the root medium is generally close to atmospheric, and by definition set to zero. The percentage contribution of the CTC and apoplast (apo) path to the total uptake of water and ions depends on several factors. These factors can stimulate, reduce or have no clear effect on the flow through each path. Those factors which are of relevance here are salt stress and the transition between day and night.

To take up water from soil, roots such as in the monocots barley and wheat need to transport water radially through different cell layers from the epidermis, across the cortex, past the endodermis and into the stele where the xylem vessels are located [138,150,151]. There are three different paths which water can take along that route: (i) the apoplastic path where water moves along cell walls and extracellular space between walls; (ii) the symplastic path where water moves through plasmodesmata from the protoplasm of one cell into the protoplasm of the neighbouring cell, while remaining within the ‘cytoplasmic continuum’ of tissue(s); and (iii) the transcellular path, where water crosses the plasma membrane as it enters and exits successive cells [138] (Figure 3). Water then moves either across the phospholipid bilayer or through membrane-intrinsic water-channel proteins, aquaporins (AQPs). The symplastic and transcellular paths are also referred to together as the ‘cell-to-cell’ path [138,151]. Note, that there are currently no methods available to distinguish between the movement of water along the symplastic and transcellular paths; and water which moves along the transcellular path between the innermost cortex cell layer and adjacent endodermal cell layer has to deal with the hydrophobic suberin lamellae, which are located between the wall and plasma membrane of endodermal cells [152,153]. That means that water transport which is facilitated entirely through AQPs between the root epidermis and stele still encounters a potentially large resistance in the apoplast in the form of suberin lamellae.

The biophysical force for water movement across the root cylinder is a difference in water potential, Δψ, between the root xylem and root medium (less negative ψ) (Figure 3). The ψ of xylem is dominated by two components, the tension, or hydrostatic pressure component, and the osmotic component. It is generally assumed that the tension component dominates during the day and is negligible during the night, when the osmotic component dominates. However, this may not always apply. Data on xylem tension during the night are hard to come by in the literature. Why should xylem in plants which are exposed to a low-ψ environment in the soil such as during drought and salinity not also have significant tensions during the night, particularly when these plants show significant rates of night-time transpirational water loss? Relying entirely on osmotic forces on water uptake during the night requires energy for the solute loading of the xylem (drought) or for dealing with high loads of salt (salinity). Accordingly, we observed in a recent study on salt-stressed wheat that the xylem was also under significant tension during the night [77,154]. More studies are needed to verify those data on other species and for a range of environmental conditions.

It is generally considered that the apoplast displays no semipermeability and that the reflection coefficient for solutes, σ, such as Na^+^ and Cl^−^, approaches 0; in contrast, σ for a near-perfect semipermeable membrane approaches 1.0. The σ of an entirely apoplastic flow path across the root cylinder should also approach 0, whereas σ of an entirely cell-to-cell path should approach 1.0 [138]. Hydrostatic pressure gradients which act along the apoplast cause ions to move by mass flow in the solvent ‘water’, whereas water movement along the cell-to-cell path is by osmosis. When we add salt to the root medium, we lower the ψ of the medium by about 0.1 MPa for every 20 mM of NaCl (40 mM of solutes), but only if we deal with a perfect osmotic system (σ = 1.0). This leaves us with the somehow paradoxical situation that, if we had a root system which lacks any semipermeability (σ = 0), the plant would not experience any osmotic stress, yet it would take up salt in an uncontrolled way along the apoplast by mass flow. If we had, on the other hand, a root system with perfect semipermeability (σ = 1), the plant would experience the maximum osmotic stress, yet would be able to filter out Na^+^ and Cl^−^ as well as the properties of plasma membrane permit. We do not know whether plants can regulate σ in the short term, for example through modification of membrane transport properties, but such a regulation would ideally be suited to deal with any short-term osmotic shock while minimising longer-term ion toxicity.

### 3.3. Root Water Uptake—Hydraulic Conductance and Hydraulic Conductivity

It has been generally considered that the radial transport path across the root cylinder rate-limits the water uptake by a root system, as opposed to the axial transport path along xylem conduits [155]. Recent work suggest that this may not always hold, and one needs to ask the question why xylem vessels should be wider than required, considering that water under a given tension becomes more metastable within increasing vessel diameter [156]. The radial rate of water uptake by a cylindric root, and a root system which consists of many cylindric roots, increases in direct proportion with the driving force between the xylem and root medium and the surface area perpendicular to the direction of flow. Hydraulic conductance, L, describes the rate of water uptake per unit of driving force (m^3^ s^−1^ MPa^−1^); when L is related to root surface area, we obtain the surface-independent size, hydraulic conductivity (Lp). It is Lp which reflects best the intrinsic water-transport property of a root [157]. In contrast, L is better suited to assess the contribution of different parallel transport paths to the water uptake of a root system. This is because L of parallel paths such as the apoplast and cell-to-cell path is additive; and the inverse of L, the hydraulic resistance, is additive for elements, or cells, which are arranged in series, for example, different tissues across the root cylinder [138,151].

### 3.4. Root Water Uptake—Regulation of the Cell-to-Cell Path Involving Aquaporins

Aquaporins (AQPs) make up most of the hydraulic conductivity (Lp) of root cells, at least those root cortical cells which have been studied in most detail [158,159]. Studies which showed the importance of AQPs for root and root cell Lp mostly applied inhibitors such as HgCl_2_ and H_2_O_2_ of AQP function or used transgenic approaches [154,158,160,161]. Cell Lp can be controlled by the number of active AQPs, either through trafficking, gating, heteromerisation or at the translational and gene transcriptional level [161]. For example, the accumulation of AtPIP2;7 in the plasma membrane was reduced by TSPO-related protein in response to abiotic stress in *Arabidopsis* [162]. *Arabidopsis* generally downregulated plasma membrane intrinsic protein (PIP), AQP transcript level and protein level in response to drought stress. Salt stress and exogenous application of salicyclic acid (0.5 mM) inhibited root Lp through internalisation of AtPIPs and removal from the plasma membrane [163]. Treatment of plants with 100 mM NaCl induced a rapid (half-time, 45 min) and significant (70%) decrease in Lp through changes in aquaporin gene expression, which may have involved a coordinated transcriptional down-regulation and subcellular relocalisation of both plasma membrane and tonoplast-localised AQPs (PIPs and TIPs; [164]. Aquaporin gating involves the opening and closing of the AQP water channel pore through phosphorylation and dephosphorylation [161]. Rapid (min) changes in root Lp following excision of root systems or reduction in transpirational water loss are associated with rapid decreases in the gene expression of AQP isoforms in a range of plant species [165,166,167]. A possible signal which controls the expression level of AQPs in root cells is xylem tension, though we do not know currently the precise mechanistic basis for this link [165].

A general response of roots to salt stress is that Lp decreases, be it either in response to the sudden application of salt stress (osmotic shock) or as part of a longer-term response [74,154,168,169,170,171]. The decrease in root Lp is associated with a decrease in the Lp of root cortical cells, though we do not know to which extent the Lp of other tissues, particularly the endodermis, changes as well [158]. In a recent study on hydroponically-grown wheat, we tested whether the reduction in root Lp is also observed during the night period, as there was a complete lack of data on this aspect. Reductions in root Lp in response to salt stress during the night were smaller compared with those during the day when comparing salt treatments with unstressed control plants. This was mainly because of an overlapping effect of the dark period on the root Lp in non-stressed plants: because Lp decreased here during the night, salt stress could not reduce Lp much further [154]. The changes in root Lp were accompanied by a decrease in the gene expression level of some but not all of the AQP isoforms tested [154]. It appears from that study that salt stress reduces root Lp during the day to some base level. This base level is also approximated during the night in non-stressed plants. As a result, root Lp does not differ a lot between salt-stressed and non-stressed plants during the night, even though plant transpirational water loss can still be significantly lower in salt-stressed plants [154]. More studies are needed to test this idea on a range of plant species. The base level of Lp which cannot be reduced further through salt stress or through a day-night transition could reflect water transport by simple diffusion across the membrane lipid bilayer, through plasmodesmata or along the apoplast. We look at the latter two processes in the next sections.

### 3.5. Root Water Uptake—Water Flow through Plasmodesmata

The big unknown component in transcellular transport of water through plant tissues is plasmodesmata (PD). This is because we cannot currently distinguish between water transport through the lipid bilayer or aquaporins (AQPs) and transport through PD. Inhibitors of AQP activity such as H_2_O_2_ also inhibit PD function, and the same may apply to HgCl_2_ [172]. It needs to be seen to which extent recently tested inhibitors of PD function, such as chitinosan oligomers in studies which focused on pathogen stress, also inhibit root Lp [173]. Most of these inhibitors cause a change in callose formation around the PD sleeves, leading to a constriction and reduction in diameter and conductance. One tissue location where PD could have a key role in affecting root Lp is at the boundary between the innermost root cortical cell layer and the endodermis. As suberin lamellae impact here negatively on water transport through, e.g., AQPs, PD offer the only known gateway to avoid this apoplastic resistance. Let us therefore have a closer look at some of the studies which have focused on PD in roots.

Plasmodesmata are plasma-membrane-lined tubes, or tunnels across the cell wall, which generate cytoplasmic and PD continuity between neighbouring cells [174]. A generic PD has two major components: membranes and spaces. Membranes form the boundaries of the PD channel through which transport may occur. The plasma membrane between adjacent cells defines the outer limit of the PD. The axial centre of the PD is formed by the desmotubule; it is a rod-like structure which is derived from the endoplasmic reticulum, transverses the centre of the PD and results in thin cytoplasmic spaces or cytoplasmic sleeves. This space is occupied by cytoskeletal proteins that contribute to channel function [175]. The cytoplasmic sleeve of the PD is the main route for intercellular molecular trafficking. The diameter of PD differs between tissues, for example, the diameter in meristematic and young tissues is 25–45 nm and in more mature tissues in the range 50–60 nm [176]. The difference in diameter seems to be due to differences is the size of the gap between desmotubule and plasma membrane [177]. The structure of PD ranges from simple, characterised by a single sheath of cytoplasm, to complex, characterised by branched, H-shaped, and twinned structures [175,177]. These structures also appear to be developmentally regulated, as young tissues commonly have simple PD, with complex PD arising later, after cell expansion has been completed [178]. Plasmodesmata with complex structures may allow higher rates of transport through them [177].

In phloem cells typical branched PD can be found. This type of PD may also exist through the walls of all root cells from the cortex to the xylem parenchyma [179]. Water transport through PD could be regulated by adjusting the number of PD per unit wall area (frequency) or the size of the PD pore. The number of PDs differs in developmental zones of a root. Most, or all cells in the root meristems are connected at a high frequency of PDs, and density decreases as cells mature [180]. For example, in Arabidopsis roots, undifferentiated epidermal cells in the meristem and elongation zone are extensively connected through PDs, as measured by dye coupling, but become less connected as they mature, until in the mature root, epidermis and root hairs are entirely isolated from the cortex cell layer beneath [181]. The distribution of PDs also differs radially across the root cylinder. Ma and Peterson [182] studied PD frequency in *Allium cepa* L. roots, at about 100 mm from the tip, where the roots had formed a mature exodermis. The frequency of PDs was high at the interfaces of the exodermis–central cortex, central cortex–endodermis and endodermis–pericycle (4.05 × 10^5^, 5.13 × 10^5^, and 5.64 × 10^5^, per mm root length, respectively). Two interfaces had a particularly low PD frequency: this was at the epidermis–exodermis (8.96 × 10^4^) and pericycle–stelar parenchyma (6.44 × 10^4^ per mm root length) transition. In the pericycle, the radial walls had a high PD frequency, a feature that could permit lateral circulation of solutes and facilitate ion (inward) and photosynthate (outward) delivery. The pore of PDs can exist in three states, open, closed and dilated [183]. The open state enables molecules of up to 1 kDa in size to pass through, whereas the closed state prevents any movement. The dilated state allows larger molecules (30–50 kDa) to pass through, as observed particularly in growing tissues. Deposition of callose, a cell wall polysaccharide, can regulate the aperture of the PD pore. Callose has been known to be deposited adjacent to the PD’s neck region. An increase in callose pushes the plasma membrane inwards and closes over the cytoplasmic sleeve, the aperture of the PD therefore decreases. Mutations and conditions that affect the metabolism of callose at PD significantly modify PD transport capacity. The enzymes beta-1,3 synthase and glucanase degrade callose surrounding PD and increase the size exclusion limit of plasmodesmata. This activity negatively affects PD aperture [184]. A range of stress signals induce PD closure via callose deposition including cold, wounding, and reactive oxygen species [184]. The most recent example is salicylic acid (SA), which regulates the formation of complex PD and callose biosynthesis to influence symplastic transport. Salicylic acid seems to activate the expression of PDLP5 (a receptor like kinase), which modifies the activity of callose metabolic enzymes to regulate symplastic transport and pathogen infection [185]. Pectin composition in the cell wall region around PD appears to be important as well to maintain PD shape and function, and thus the permeability of PD channels [186].

There exists very little information about how PD water-transport activity or structure changes during root development or in response to abiotic stress in plants. Osmotic stress caused changes in the size of the PD channel in cortical cells of the root tip [187]. Hukin et al. [188] using the AQP inhibitor HgCl_2_ concluded for maize that most of the water transport through tissues in the meristematic tip region of roots occurred along the symplasms, and therefore involved PDs, whereas water transport in more mature tissues involved mainly AQPs. As we said at the start of this section, PDs are the big unknown in our models of how plants regulate the uptake of water by the root system. This applies to water uptake along a developmental gradient, in different types of roots (e.g., seminal, adventitious, lateral), and in response to stress. Similarly, we can currently only speculate how much PDs may contribute even to fast (min) responses of root Lp to stress. Given the location of suberin lamellae and their potential obstruction of transportation of any molecules, not just water, along a membranous path between the innermost cortex cell layer and endodermis, we can predict that PDs must have a key role in regulating how much of a molecule arrives in the stele and is transported to the shoot.

### 3.6. Root Water Uptake—Water Flow along the Apoplast, and ‘Bypass’ Flow

The main barriers to water and solute movement along an apoplast path are suberin lamellae and, particularly, Casparian bands [141,154,189]. These form at the endodermis, but Casparian bands especially can also result in the formation of an exodermis, beneath the root epidermis [140]. For example, under water stress, species with an exodermis (e.g., *Zea mays*, *Helianthus annuus*, *Alliums cepa*) showed less water loss into the root environment compared with species which lacked an exodermis (e.g wheat, *Pisum satium*, *Vicia faba*, barley) [190]. The endodermis was considered to be an important regulator of hydraulic conductance across soybean varieties, but this was more related to the dimension of the endodermis rather than degree of suberisation [191]. Sutka et al., (2011) [192] found no consistent relationship between the suberisation pattern and root hydraulic conductivity across five *Arabidopsis* accessions. Ranathunge and Schreiber (2011) observed for *Arabidopsis* mutants with altered suberin composition that a reduced tissue content of certain suberin polymers was associated with an increased radial hydraulic conductivity [153]. Suberisation and lignification of the endodermis have often been observed to increase in stressed roots [140,154]. For example, the biosynthesis and deposition of suberin was stimulated in primary roots under drought and salt stress [193,194], and increased biosynthesis of suberin was associated with increased drought tolerance in grape, though suberin layers in fine roots may have reduced tolerance. This could suggest that the effect of suberin on drought tolerance is root-type specific [195]. Wild barley has more suberin deposited in the exodermis under drought compared with cultivated barley, similar to other species adapted to drought [196,197]. In drought-stressed rice, suberisation of the endodermis increased, whereas suberinisation of the sclerenchyma layer decreased. This could increase the retention of water [198]. Suberin has been shown to aid the exclusion of salt through a barrier function in the endodermis [153,199]. The deposition of lignin around xylem vessels has been reported to increase drought tolerance, by making the region next to conducting xylem elements less permeable to water [200,201]. Expression of MYB41 is upregulated during drought and salt stress and by abscisic acid (ABA). This has been related to an increase in the biosynthesis and deposition of suberin in *Arabidopsis* and grapevine during salt stress and a reduced radial water loss by roots [202]. In addition to lignin and suberin lamellae, other factors, such as lysed cortical cells and the formation of aerenchyma, could alter the apoplastic resistance to water movement [203].

‘Bypass flow’ is a term that has been used to describe the leakage of water and in particular Na^+^ and Cl^−^ across the main apoplastic barriers in roots [137,204]. Bypass flow has been studied mostly in the context of salt stress [137,204,205]. The best studied species is rice [137,204,205]. Bypass flow refers in most cases to the apoplastic leakage across the endodermis, as this tissue forms an integral part of the root structure and undergoes various defined stages of development during which Casparian bands, suberin lamellae and thickened inner tangential walls are formed in succession [137]. The exodermis presents another apoplastic barrier in roots yet, unlike the endodermis, the exodermis is not a tissue per se. Rather, the term ‘exodermis’ refers to the modification of the tissue layer beneath the epidermis in response to stresses such as drought and salinity, or flooding, where radial oxygen loss needs to be minimised [140]. The formation of exodermis involves the deposition of Casparian bands and, occasionally, suberin lamellae [140]. A third apoplastic barrier, which has received less attention, is the heavy lignified apoplast of the root stele, where xylem parenchyma occupies most space [206].

Bypass flow of ions such as Na^+^ and Cl^−^ in roots of salt-stressed plants occurs by mass flow; it is driven by a difference in hydrostatic pressure between root stele (xylem) and root environment. The main driver during the day is xylem tension. The higher the xylem tension is (the more negative xylem hydrostatic pressure is), the larger the potential bypass flow of ions into the stele and subsequent delivery to the shoot. We would expect that bypass flow decreases during the night, when xylem tension is lower or absent. This is indeed the case, as we observed recently in a first study of its kind on salt-stressed wheat (Lu and Fricke, in preparation). One could argue now that these wheat plants experience less ionic stress during the night, which is in some ways correct, as less Na^+^ and Cl^−^ leaks into the xylem. However, that same study also showed that water uptake along the cell-to-cell path decreased relatively more during the night than the bypass flow of ions decreased. That means that the molar ratio between taken-up water and ions decreased during the night. In other words, a more concentrated salt solution arrived in the shoot during the night.

The major cereal crops, barley, wheat, maize and rice, show a decreasing tolerance to salt in that sequence [1,207]. Barley and wheat have a root reflection coefficient ‘σ’ for Na^+^ and Cl^−^ of close to 1.0. This points to near-perfect semi-permeability. The σ in rice and maize is significantly smaller than 1.0 [150,158,160,208,209]. Accordingly, the highest %-bypass flow of water and ions of these four cereal crops occurs in rice. Rice showed 5% to 30% bypass leak of Na^+^ when grown at 50 mM NaCl, that is between 5% to 30% of the total Na^+^ delivered to the shoot had leaked through the root apoplast. Two wheat cultivars, which were analysed as part of the same study, showed bypass leaks of Na^+^ of only 2.9% and 3.3% [137,204,210,211]. The high bypass flow of Na^+^ and Cl^−^ in rice has been linked to its poor salt tolerance, and there exists a negative correlation among rice genotypes between tolerance to salt and the amount of bypass flow [210]. We need to remind ourselves that rice still takes up 70% and more of the total Na^+^ (and Cl^−^) delivered to the shoot along the membranous, cell-to-cell path. Should it not be possible for plants to down-regulate the flow rate of these ions along this path, for example through reduced activity of uptake and increased activity of export transport systems? Also, a crop such as wheat, which takes up less than 3% of the total Na^+^ and Cl^−^ along a bypass route, is more salt-tolerant compared with rice, but it still suffers yield reductions at salinities of 100 mM and more [3]. Is it really the salt taken up along a bypass path that matters to salt tolerance, or is the bypass flow of ions rather a consequence of a property of endodermis that impacts salt tolerance through some other mechanism? We do not have an answer to this question. The most likely mechanism which comes to our mind is xylem tension and embolism. Could it be that the apoplastic seal around the endodermis reduces the likeliness that air is drawn from intercellular air spaces within the root cortex and seeds embolism in the stele and xylem? One would expect that the narrow capillary diameter of xylem parenchyma walls prevents this from happening, but we do not know for sure. In addition, endodermal development progresses along the root axis, so that most mature zones have a completely developed endodermis—and xylem tension in these zones is largest and closest to tension in the shoot.

## 4. Conclusions

We focused in this review on the water and solute relations of plants which are exposed to salt stress. We also raised the question of what we know, or don’t know, about processes which occur during the night. Our ideal crop plant which can yield to 100% at salt concentrations exceeding 100 mM, or maybe 200 mM NaCl, remains elusive! This is not so much because of the lack of mechanisms which exist across the plant kingdom to deal with the major stress components of salinity. It is rather because of the additional energy requirements that are associated with those mechanisms. This additional energy leaves us with a catch-22, grow as much as before while spending more energy, but do not increase significantly carbon and energy assimilation. Even if the energy question was solved, plants may still be left with physical, or chemical, limits, particularly as concerns xylem tensions which can be tolerated without embolism and the co-existence of several chemical substances within cells at upper mM concentrations. We are left with the apparent paradox that bypass flow and leakage of Na^+^ and Cl^−^ into the root stele can be related to salt tolerance in a crop such as rice, yet even this crop takes up most Na^+^ and Cl^−^ along a membranous path which can be down-regulated in its transport activity.

A common response of plants to salt stress is a reduction in root hydraulic conductivity (Lp). This reduction is achieved through a reduction in the activity of aquaporins (AQPs) and involves a range of regulatory means. Again, we need to ask why root Lp is actually reduced in response to salt stress, particularly during the day period? Most water is taken up along a (cell-to-cell) path which allows the selective uptake of water and retention of Na^+^ and Cl^−^. Xylem tension increases during the day in response to salt stress [154], and an unchanged root Lp would result in an increased rate of water uptake along a highly water-selective path, provided root surface does not decrease. Plants could then afford to lose more water, which in turn would enable more gas exchange and assimilation of carbon and fixation of chemical energy. Why then, does root Lp decrease? We do not have a final answer, as potential loss of water to the saline root environment is not a problem, since plants take up net water during day and night to support growth in the leaf area. Stomatal closure to reduce plant transpirational water loss cannot be an explanation either, as this reduces xylem tension and, through this, the driving force for water uptake and water uptake as such—without any required reduction in root Lp. Maybe the uptake of water along the cell-to-cell path and the movement of Na^+^ and Cl^−^ along this path are not as much uncoupled from each other as we think; or there are features and functions associated with aquaporins (AQPs), such as their high membrane abundance [212] which drains resources, that we are not aware of or have been studied little (Na^+^ transport properties [213]) and which govern the response of plant to salt stress. Notably, root Lp generally decreases in response to stress [74]. This implies that root Lp and AQP-facilitated water transport operate already at or near the maximum level in non-stressed plants, and the number of AQP molecules which can be fully functional or inserted per unit area of the plasma membrane may be limited. The future will show.

We would like to end this concluding part with highlighting the key findings and questions of this review, and our own personal views on these:

- There exists a large gap in our knowledge of the contribution of night-time-related processes, not just to the tolerance of plants to salt stress but to the functioning of plants in general.
- We were surprised to see how little information there is on xylem tension during the night, for any plant species under any environmental conditions. Plants grow in size during the night and need to take up considerable amounts of water as well. They can also transpire significant quantities during the night through partly-open stomata. Surely, there must also be significant xylem tensions during the night, particularly in a low water-potential soil environment, in some species.
- Maybe the largest unknown player in the regulation of the transportation of water and ions across tissues is the role of plasmodesmata. This is not so much because we do not think that they are important. It is more because we cannot study their transport properties in isolation, nor can we quantify the flow rate of water and ions through them. We tend to neglect aspects or assume that they play a minor role, as in the case of plasmodesmata, when we have no means to study them in detail. This renders the modelling of flows easier, but it does not make this modelling reflect more accurately the true situation in a plant.
- It is tempting to search for and identify molecular stress tolerance mechanisms and use these for targeted breeding. However, unless these mechanisms save energy and carbon to plants, they are likely to fail. This applies more to the agricultural context, where the focus is on optimising yield; it does not have to apply to a natural, non-agronomic setting, where the emphasis is often on survival.
- The endodermis is a barrier to the apoplastic (bypass) flows of water and ions across the root stele, from the cortex into the stele, and therefore, ultimately, root xylem and shoot. Is this really the entire story? Casparian bands constitute the main barrier to the radial movement of water and ions, but what about suberin lamellae? As the latter are located between the wall and plasma membrane of endodermal cells, they should also form a barrier to water and ions which exit the innermost cortex cell layer and want to enter the endodermal cell through a plasma membrane. Does that mean that the function of suberin lamellae could be more about forcing flow of water and ions between these tissue layers to occur through plasmodesmata; and to minimise the leakage of solutes out of endodermal cells through the plasma membrane into the cortex? We do not know. Could it maybe be that suberin lamellae provide an additional means to prevent the formation of air bubbles and embolism in the stelar apoplast including xylem vessels? One would not expect this based on the diameter of nanochannels in the wall and plasma membrane space, but we do not know for sure. Nor do we know how much of the tension in the xylem and stele transmits into a tension in the root cortex.
- We predict here that the contribution of night-time-related processes to the tolerance of plants to salt stress and their productivity in general is far more important than we anticipate today. We also predict that this importance varies with species, soil water potential and the difference in temperature and relative humidity between day and night—sizes which are affected differentially by climate change.

## Data Availability

Data requests can be made to the corresponding author upon reasonable request.
